# A Novel Quick-Response Eigenface Analysis Scheme for Brain–Computer Interfaces

**DOI:** 10.3390/s22155860

**Published:** 2022-08-05

**Authors:** Hojong Choi, Junghun Park, Yeon-Mo Yang

**Affiliations:** 1 Department of Electronic Engineering, Gachon University, 1342 Seongnam-daero, Seongnam 13306, Korea; 2 School of Electronic Engineering, Kumoh National Institute of Technology, 61 Daehak-ro, Gumi 39177, Korea

**Keywords:** motor imagery classification, eigenface analysis, quick response neuro images, image data augmentation, standardized and sharable quick response eigenfaces

## Abstract

The brain–computer interface (BCI) is used to understand brain activities and external bodies with the help of the motor imagery (MI). As of today, the classification results for EEG 4 class BCI competition dataset have been improved to provide better classification accuracy of the brain computer interface systems (BCIs). Based on this observation, a novel quick-response eigenface analysis (QR-EFA) scheme for motor imagery is proposed to improve the classification accuracy for BCIs. Thus, we considered BCI signals in standardized and sharable quick response (QR) image domain; then, we systematically combined EFA and a convolution neural network (CNN) to classify the neuro images. To overcome a non-stationary BCI dataset available and non-ergodic characteristics, we utilized an effective neuro data augmentation in the training phase. For the ultimate improvements in classification performance, QR-EFA maximizes the similarities existing in the domain-, trial-, and subject-wise directions. To validate and verify the proposed scheme, we performed an experiment on the BCI dataset. Specifically, the scheme is intended to provide a higher classification output in classification accuracy performance for the BCI competition 4 dataset 2a (C4D2a_4C) and BCI competition 3 dataset 3a (C3D3a_4C). The experimental results confirm that the newly proposed QR-EFA method outperforms the previous the published results, specifically from 85.4% to 97.87% ± 0.75 for C4D2a_4C and 88.21% ± 6.02 for C3D3a_4C. Therefore, the proposed QR-EFA could be a highly reliable and constructive framework for one of the MI classification solutions for BCI applications.

## 1. Introduction

The brain–computer interface (BCI) is a continuously developing technology that implement the brain’s thought to manipulate external bodies without thinking through the human nerves to the hands or feet [1,2]. Thus, BCI systems are intended to generate new communication channels for other human parts [3,4]. There are two types of justifications available. One is uni-directional communication from a brain to a computer and the other is bi-direction communication between a brain and computer, as well as brain-to-brain. The current state of art technology for the BCI is bi-direction BCI technology or brain to brain interface (B2BI) [5]. The B2BI functions via on one hand as a brain–computer interface (BCI) to retrieve messages and, on the other hand, a computer-brain interface (CBI) [6]. The communication paths that do not go through human’s nerves could help people with physical disabilities by controlling objects, such as a wireless mouse [7]. The signals that are generated from the human brain are used to implement the systems, and they are extremely complex; such signals are recorded in electronic system, i.e., an electroencephalogram (EEG) [8]. In the beginning of EEG research, EEG measurement used invasive and painful methods, in which several electrodes were inserted directly into the human skin [9]. The non-invasive methods of attaching the electrodes to the human scalps have been used because they are non-painful, user-friendly, and economical ways to obtain EEG signals [10]. In particular, the classification of motor imagery (MI) is researched because EEG signals are shown when the right and left hands are related, respectively [11].

**Figure 1:**
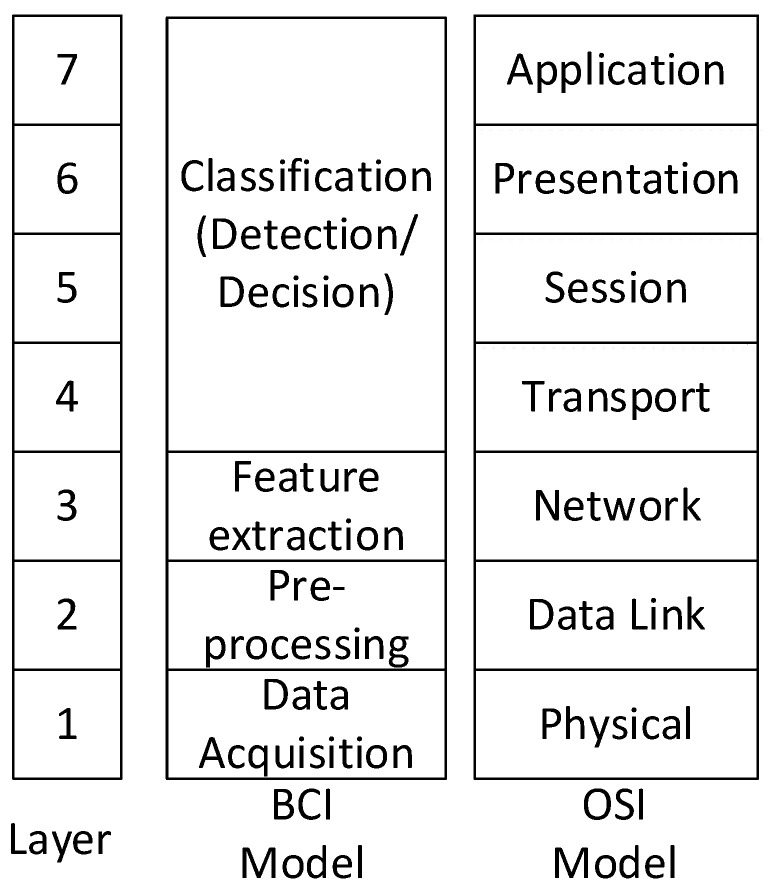
The BCI layer model compared to OSI network model.

In the BCI systems, raw EEG signals need to be processed with filtering, extraction, and classification techniques [12]. We describe the whole BCI workflow based on the OSI 7-layer model; the OSI 7-layer model is a kind of the communication model. Therefore, it could be useful for readers to understand the feature extraction and classification algorithms in the signal processing workflow. In OSI 7-layer model, there are two types of disciplines. One is a datagram for a layered network hierarchy, and the other is a conceptual model for multistage processing. In the layered hierarchy model, there are the physical, medium access control, network, transport, or routing, etc. In the conceptual model for multistage processing, the focus is on multistage processing, such as the input, data acquisition, preprocessing, decision/detection, and hypothesis test. In this article, based on the previous literature of IEEE [11], we compared the similarity between the BCI dataflow and OSI 7-layer in the conceptual model for better understanding of the signal progressing in BCIs. According to N. Khadijah et al., in the previous related work [13], we used the open system interconnection (OSI) layer, as shown in Figure 1. In the BCI model, we used a layer architecture staring from data acquisition (layer 1), going through pre-processing (layer 2), approaching at feature extraction (layer 3), and then ending at classification (layer 4).

In the signal processing steps, feature extraction was used; generally, a filter block is applied before feature extraction [14,15,16]. There are several feature extraction methods, such as wavelet transform, short-time Fourier transform (STFT), common spatial pattern (CSP), regularized CSP (RCSP), common spatio-spectral pattern (CSSP), common sparse spectral spatial pattern (CSSSP), etc. [8]. As a representative visualization method, short time Fourier transform (STFT), spectrogram, wavelet, etc., were used [17]. Among those methods, the CSP is one of the most widely accepted feature extraction methods [18]. However, the CSP method has overfitting problems when data are sparse in the domain. The CSP method is also noise sensitive, so it is difficult to classify multi-class EEG signals. Blankertz et al. focused how a CSP filter affected the EEG signals [19]. The combination of CSP has been used by many researchers, including Lotte and Guan [20]. Their idea was used to regularize CSP and substitute the normal CSP. The limitation is that the researchers were required to use the full set of data for the training and testing sections. The training data should have a larger number of data than the test data. The full set of data that were used, especially the test data, may lead to an overestimation in performance because all the information is being used. To classify the MI for a subject, the algorithms required the use of other subjects, as well. Therefore, the algorithms are not suitable for classifying a single subject dataset. The extended algorithm of the CSP was used by Lemm et al. [10]. This algorithm is known as CSSP. The CSSP method introduced a delay to the system, so that the spectral filter was included in the system. The simulation results, except for six datasets, showed better accuracy than CSP.

In current EEG classification studies, linear discriminant analysis (LDA), support vector machine (SVM), deep learning, etc., were used [21]. Deep learning (DL) is a machine learning (ML) method used in various fields, such as the voice and video processing fields [22]. The DL works successfully on non-linear and non-stationary data, and it works efficiently even in the fields that are difficult for humans to distinguish [23]. The convolutional neural network (CNN) is one of the DL algorithms that is widely used for data classification [24]. Due to CNN characteristics, there are attempts to apply CNN for EEG signal classification. To use the CNN method specialized for image classification, various methods to visualize EEG signal are being studied [25]. Visualization of these EEG signals was used to help improve the classification performance of DL models using CNN algorithms. Compared to those traditional classification algorithms, such as LDA and SVM, the deep learning model requires the use of large numbers of the dataset [25]. Therefore, the limited numbers of the dataset need to be increased using a data augmentation technique. Considering the finite training data in BCIs, Huang et at.al proposed a data augmentation scheme via a CNN [26]. The data augmentation has accomplished by mixing and recombining the images. Lee et al. convers the lingering problem of zero-training by utilizing a proposed CNN model connected to P300 information [27].

In the EFA method, the calculated eigenface coefficient was used as a feature for data classification. In this case, it is possible to reconstruct the pictures using the eigenface coefficients. We proposed a novel EEG signals classification method, called quick response EFA (QR-EFA) utilizing deep knowledge in standardized and sharable QR image formulations. The QR-EFA is subjected in the EEG signal preprocessing stage. Image reconstruction or data augmentation using QR-EFA has been shown to generate QR image features suitable for CNN algorithms. To overcome the constraints, such as the trial numbers and limited BCI competition dataset in EEG, we proposed an innovative classification technique that was organically designed for EFA and CNN based on data augmentation. After learning the impulse response filter for the BCI competition IV dataset 2a (C4D2a_4c) [28] and BCI competition III dataset 3a (D3D3a_4c) [29], using a seven-layer simple CNN model (one input and output, one convolution, one pooling, and three fully connected), the test EEG data classification performance was measured according to the transfer function. As of today, the highest classification results for EEG 4 classes on BCI competition IV 2a is 85.4% [1]. Based on this observation, our proposed QR-EFA method could provide higher accuracy performance of MI classification for competition dataset in BCIs.

## 2. Materials and Principles

The proposed EFA algorithm is a feature extraction method from EEG data that builds up neuro images, emphasizing the discriminability of classes; the feature is a kind of tool.

**Figure 2:**
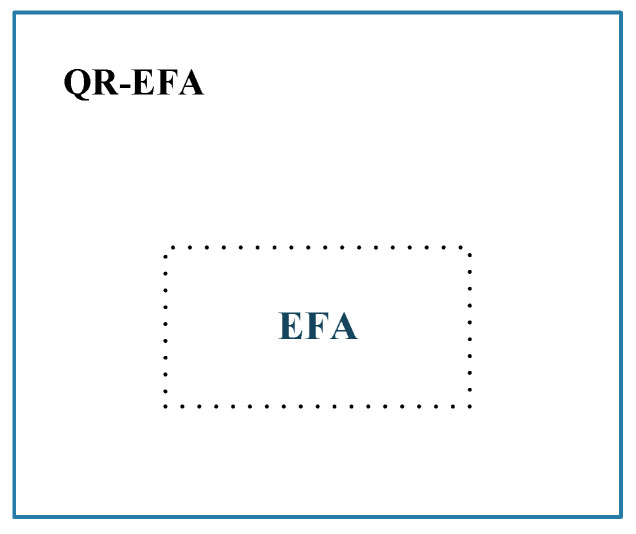
Overall collaboration between QR-EFA and EFA.

The QR-EFA method was formulated based on the previous EFA result. From egienfaces derived from the previous EFA, we will provide the description. First, the eigenface is asymmetric in horizontal and vertical directions, and the image size is excessively bigger than the size that CNN can process. Second, in BCI competition dataset, there are many discrepancies in data sizes among number of classes, trials, and subjects. Based on this fact, we proposed QR eigenface to provide a symmetrical, standardized, and sharable sizes in images. In the framework level, QR-EFA consists of the parts, such as EFA, eigenface restructuring, data augmentation, and CNN. Figure 2 shows the relationship between the previous proposed EFA and proposed QR-EFA.

**Figure 3:**
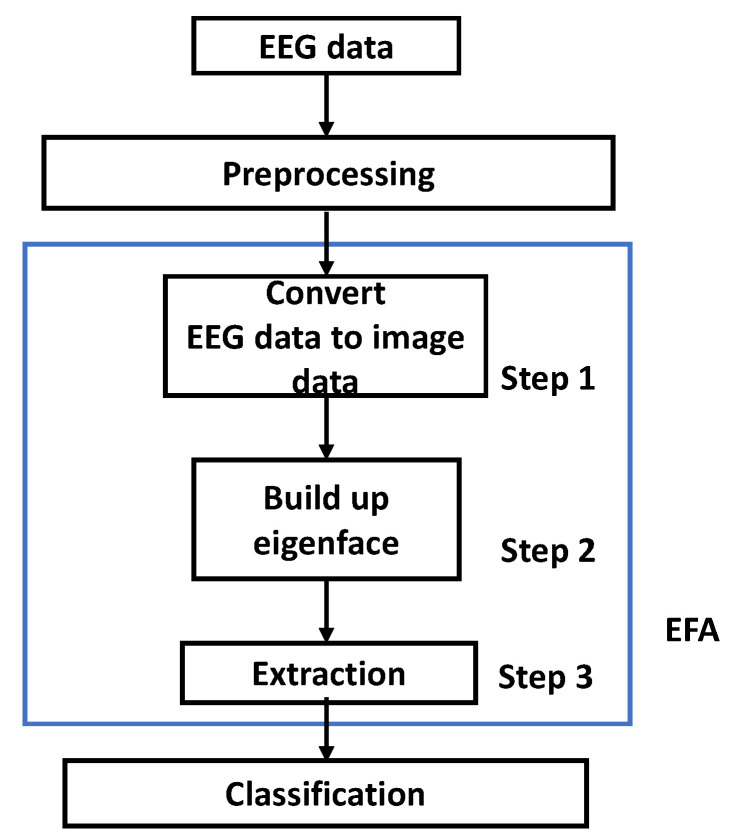
The EFA algorithm procedure.

The EFA is different PCA image recognition type for dimension reduction [30]. The fundamental EFA method is depicted in Figure 3. The EEG data were preprocessed. As shown in steps 1–3, EEG data were converted to image data to build up eigenface. From eigenface, coefficients, which we called features, were extracted for training and testing procedures. Afterwards, the classes were classified for the next step.

Let us define the next step of the data interpretation after EFA procedure. We also used the same data interpretation techniques for classification.

Step 1: The three-dimensional (3D) converted EEG image data could be separated as time, channels, and trials. The data were recognized in each separated 3D converted EEG image data, and the generated data could be differentiated, depending on the viewpoint for each 3D direction.

Step 2: From the differentiated 3D EEG image data, the covariance matrix must be obtained. Afterwards, we could determine and build up the eigenfaces.

Step 3: The training data were projected to obtain the requested features. The dataset was composed of, or represented with, ‘*time (S)*’, ‘*channels (C)*’, and ‘*trials (N)*’, as described in Equation (1).

*Dataset = time (S) × channels(C) × trials (N)* (1)

**Figure 4:**
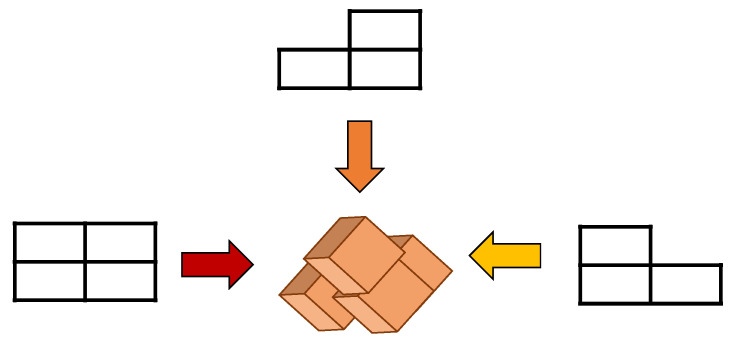
Data analysis on the viewpoint direction.

EFA is a method to obtain the differentiated EEG data with different directions. The EEG image data with the time, channels, and trials were combined into the dataset, which is an infinite number (*N*) of the image data with the same manner as that illustrated in Equation (2). In other words, we consider 3D data in two-dimensional (2D) images by combining or concatenating ‘time’ and ‘channel’ data together as in *T* = *SC* (time × channel). In fact, the derived tentative dataset, T is composed of ‘time’ and ‘channel’ components in series. The desired dataset or image matrix was obtained by rearranging the component *T* in horizontal direction and component *N* in vertical direction as shown in Figure 4. Consequently, Equation (2) shows the final dataset or 2D images.

*Dataset = TN where T = SC* (2)

The eigenface was then obtained from differentiated 3D EEG image, and newly obtained image data *Φ* and value *Ψ* need to be calculated.

*Φ_k_ =Dataset_k_ − Ψ_k_, k = 1, 2, …, N* (3)

The covariance matrix extracted from the differentiated 3D EEG image data without the mean value is obtained in Equation (4).

(4) $$C=\frac{1}{L}\;\overset{L}{\underset{k=1}{\sum }}\Phi_{k}\Phi_{k}^{T}$$

Let us define the eigenvectors of *X* with eigenvalues of *L* of the covariance matrix *C* after solving the following equation *CX* = *kX*. Among the vectors extracted from this matrix, the *k* vectors were chosen. The eigenfaces must be extracted with only training eigenface *Г_train_*. Subsequently, the training features were extracted from the training eigenface and data. The extracted eigenface coefficients were projected in Equation (5).

*Ω_train_= Φ_train_ Г_train_* (5)

The weight coefficient *Ω_train_* was obtained to be a training feature. Equation (6) shows how to obtain the feature coefficient *Ω_test_*. The eigenspace was trained, and the EEG test data can be classified as shown in Equation (6).

*Ω_test_= Г_train_ Φ_test_* (6)

## 3. QR-EFA

### 3.1. Idea Formulation for QR-EFA

The description of the procedure and flowchart for QR-EFA is shown. The proposed QR-EFA procedure is given below.

1. Perform the process with channel direction.
2. Original image eigenface formulation.
3. Confine the number of eigenfaces, up to the number of classes required and trials in common among subjects.
4. Adjust eigenface size horizontally with number of classes and vertically with number of trials.
5. Modify image eigenface formulation by multiplying a brightness factor with steps 3 and 4.
6. Obtaining training coefficients by projecting train images to the eigenface.
7. Implement QR neuro train images with the coefficients using the result of step 6.
8. Obtaining testing coefficients by projecting test images to the eigenface.
9. Implement QR neuro test images with the coefficients using the result of step 8.
10. Neuro image augmentation to diversify the limited neuro train images.
11. CNN training process with the results produced, using the result of step 10.

**Figure 5:**
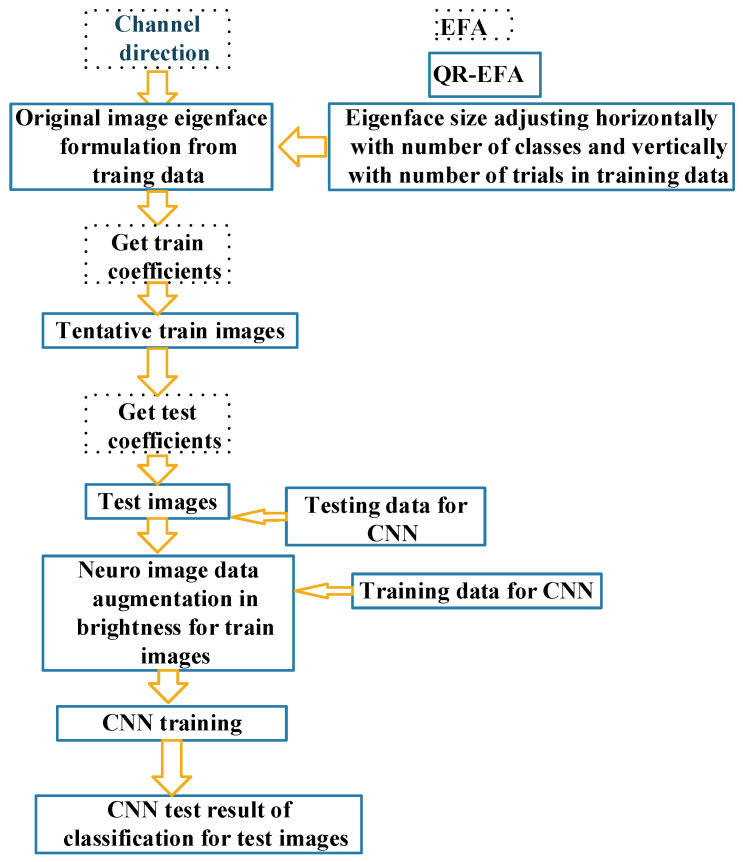
Overall flowchart for QR-EFA (solid line) including EFA (dashed line). Cross reference in the above data flowchart for step 1~11.

Figure 5 shows the flowchart of the procedure for QR-EFA, described above.

### 3.2. Data Augmentation for the Limited Training Data

After we finished the EFA method for the C4D2a_4C and C3D3a_4C datasets, the features were obtained. Based on the features, QR code-like brain neuro images will be formulated. There are two different image types. One is for the training process, and the other is for the testing process. The amount of training data set is definitely limited in BCI competitions, so we need a data augmentation process to multiply the limited images to the desired number of training images. To augment the limited training images, we adapted a brightness control for the input training images, according to the following probability model. For the given normal probability density distribution (PDF), with mean (*μ*) and standard deviation (*σ*) x ~ *N* (*μ*, *σ*^2^), the statistical random data *x[n]* will be obtained as follows in Equation (7) [31].

(7) $$\mathrm{PDF}:\;f(x)=\frac{1}{\sqrt{2\pi }\sigma }e^{-{\left(x-\mu \right)}^{2}/2\sigma^{2}},-\infty <x<\infty \;x=\mu +\sigma$$

The MATLAB code for training image data augmentation by Gaussian blur (brightness and contrast implication or image bacteria culturing per pixel) is as follows (Box 1).

Box 1 **The MATLAB code for training image data augmentation by Gaussian brightness control.**

rand_img = 1 + (0.7).* randn(is1,is2);

% 1/0.7 (example of is1,is2,is3: 18,16,30)

timg = trimages(:,:,j).* rand_img;

% neuro image culture by multiplication

The data augmentation per pixel was demonstrated to enhance the performance of deep learning approaches by reducing overfitting problems. The overfitting or high variance in ML models are produced if the dataset used to “teach” the model is greater than the testing accuracy. The overfitting problems can be generated when the model has very high errors in the testing dataset. According to the data science literature, CNN needs a huge number of neuro images for the model to be trained effectively [32]. This proposed technique could be helpful for increasing the performance of the model, thereby reducing overfitting problems from the EEG data. Most BCI competition datasets for classification and object detection datasets have a few hundred to thousands of neuro images. Considering the rule of larger numbers or the invariance property of CNN, the objects can even be classified when they are visible in different sizes, orientations, or potentials on channels. Hence, we can take the limited neuro competition dataset of images and transform the objects into different sizes by controlling for brightness, scaling in size, rotating in orientation, or zooming in and out. Through this type data augmentation, we can create intense and diverse neuro image datasets with the extent of variations.

### 3.3. Covolution Neural Network for QR-EFA

Compared to conventional classifiers, such as LDA, SVM, and multilayer perceptron (MLP), the convolution neural network (CNN) is the most widely used multi-layered neural network ML method for image classification [19]. A general CNN method learns an input image through convolution, pooling, and fully connected layers, so it acts as a classifier [33]. The convolutional layer is composed of feature maps, with several different weight vectors; each feature map is calculated from the input image. The CNN-based image classification models, including ResNet and DarkNet, are widely known [33]. Using an appropriate ML model and neural network structure, according to the target images to be classified, is very important for obtaining high classification accuracy [34]. To better show that EEG images are easily classified using the QR-EFA method, a simple CNN model using only one convolutional layer, a pooling layer, and a fully connected layer was used.

**Figure 6:**
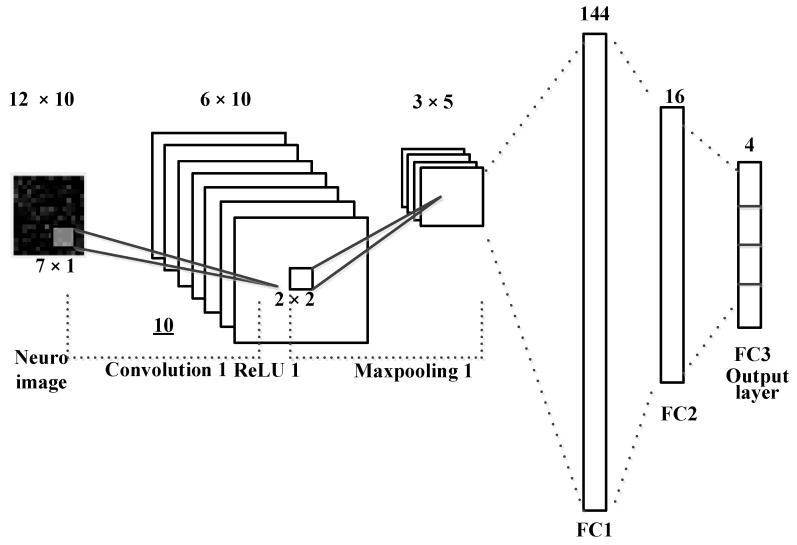
The CNN for neuro images in QR-EFA.

In Figure 6, a simple one convolution and mean pooling are shown to reduce a computation time and minimize the task burden. The size of input images is 12 × 10 and then it goes through a convolution layer with kernel or filter size of 7 × 1. After the layer process is done, it would be 6 × 10 output; then, it goes through 2 × 2 mean and max pooling layers. After processing a fully connected layer, we finally obtained the output with the class classification output for BCI dataset.

Figure 6 shows a CNN model that classifies the EEG images converted by the QR-EFA method. The convolution layer of the CNN model, which consists of sixteen filters of size 9 × 3 and a pooling layer of size 2 × 2, was used. Using softmax and cross entropy methods, two class classification results are obtained. As hyper parameters, the learning rate was set to 0.0001, and a batch size of 512 and momentum optimizer were used. The CNN model was trained for sufficient learning, and training was set to finish early to prevent overfitting problems.

## 4. Experimental Results

The QR codes are widely used for quick response as matrix barcodes. Just as with QR codes, we also obtained the images, so we called them QR images. After performing QR image data, we need further steps. In obtaining QR images for training and testing images, there are three factors to consider for better classification, in order to have accurate, robust, and reliable results. First, let us define some terminologies, such as the domain-wise similarity among domains, trial-wise similarity among trials, and subject-wise similarity among subjects. Domain-wise similarity is the degree of similarity that indicates some common characteristics on the eigenfaces between the training and testing domains. Trial- and subject-wise similarity were degrees of similarity among trials and subjects, respectively. We could recognize the similarity from QR images. Our proposed QR-EFA algorithm maximizes the similarities, with the respect to domains, trials, and subjects. As result, it could generate unique features per domain, so it is possible to discriminate the classes efficiently.

The background regarding EEG datasets from BCI competitions needs to be explained. To validate the proposed method, we used two EEG datasets, such as the BCI competition III dataset 3a (C3D3a_4C) and BCI competition IV dataset 2a (C4D2a_4C), for four classes. Specifically, C3D3a_4C is dataset from three subjects or participants, and C4D2a_4C is dataset from nine subjects, which are the off-line, publicly available, and open accessible dataset of the BCI competition database. Hence, this article focuses on the C3D3a_4c and C4D2a_4C. Table 1 shows the detailed number of trials per subject for the C3D4a_4C used in this article.

The detailed information of the property for C3D3a_4C is given as follows (Box 2).

Box 2 **The detailed information of the property for C3D3a_4C.**

comment1: ‘dataset: C3D3a_4C’

date: ‘2021.12.28’

madeby: ‘4C’

affiliation: ‘KNIT’

window: ‘offset: 3.5, length: 2’

subject: ‘subject #: 1’

prefiltering: ‘off’

s: 250 (sample/sec)

c: [1 × 60 cell]

x: [500 × 60 × 180 double]

y: [1 × 180 double]

The MI classification EEG images extracted with QR-EFA were classified for C3D3a_4C. The data augmentation using Gaussian distribution was used for sufficient training of the ML model and prevention of overfitting. From 10,800 augmented training images, which come from the data augmentation of the original QR neuro images, in brightness and without data augmentation, a total of 2592 non-augmented test images were used. As a result of the final classification experiment, the accuracy was obtained for the test dataset of 2592 sheets, as shown in Table 2.

**Figure 7:**
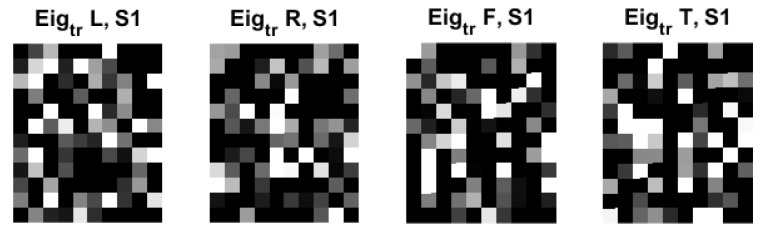
An original eigenface images of subjects for training data for 4 classes: left, right, feet, and tongue after whitening (12 × 10: = 30 × 4) in C3D3a_4C.

Figure 7 shows the examples of eigenfaces, formulated based on the QR code. Although there are lots of QR-eigenfaces are available, we will confine only a small number of eigenfaces, considering the number of classes and trials among the subjects. We chose the eigefacecs reflecting the number of classes. We designed the first to left, second, right, the third to feet, and the last to tongue classes. However, the order is subject to change as the supervisory learning constrictions. This type of decision is related to the choice regarding the approach between the supervised and unsupervised learning.

Thus far, we obtained the training coefficients by projecting the training images to the QR-eigenfaces and testing the coefficients by projecting the testing images to the QR-eigenfaces. The next step is to conceive of new QR images for training and testing the dataset. Subsequently, linear combinations of the QR eigenfaces, weighted by the relevant coefficients, were used as QR images for training and testing data.

**Figure 8:**
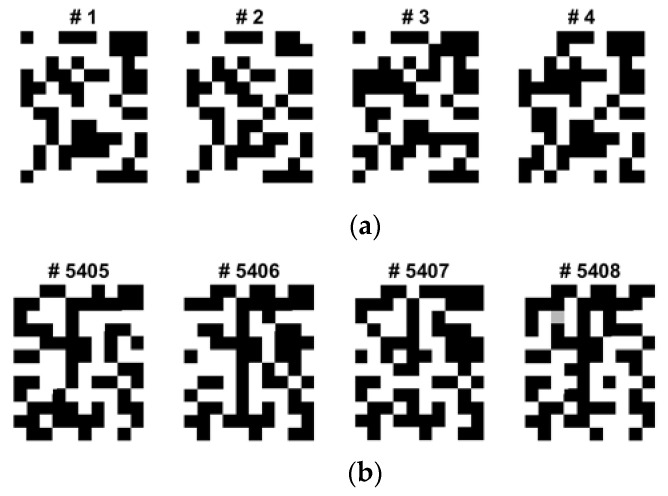
Some selected sample QR training images of data augmentation for the class of (**a**) left and (**b**) tongue (12 × 10) in C3D3a_4C.

During the data augmentation process for overcoming the limited dataset problem in the BCI competition, we modified the brightness of the recovered images based on the Gaussian noise contamination equation. Although the grayscale and quantized images from the first to last appear similar, the real binary values of the images are different and unique, and they can be used for classification. Because of ML or AI paradigm considerations, a sufficiently large number of training images are needed to train CNNs and fine-tune the logic. However, as the BCI training dataset and its QR images are finite and limited, we need to amplify or diversify them by utilizing data augmentation in the brightness direction. Figure 8 shows one of the EFA QR code implementation results after the brightness data augmentation. Among four classes, considering its dominance and the activation region of the brain, we only selected and data augmented four QR training images for the left and tongue classes, as shown.

**Figure 9:**
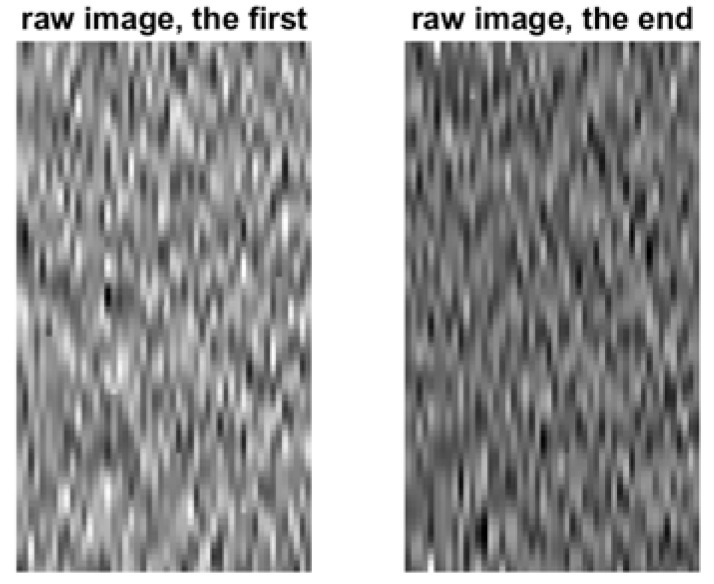
Primitive raw images for EEG data signal for the selected first and last trials (80 × 60) in C3D3a_4C.

Because the BCI data are random signals, they must be treated in statistical signal-processing domains. Figure 9 shows the typical neuro raw images considered for the QR-EFA. The left image is for the first trial, and the right image is for the last trial.

Based on the QR-eigenfaces for left, right, feet, and tongue, we proposed considering domain-, trial-, and subject-wise similarities. The detailed considerations are as follows. First, subject-wide similarities among the subjects were checked. Second, domain-wise similarity between the training and testing domains was considered. Finally, a trial-wise similarity was investigated among the trials.

**Figure 10:**
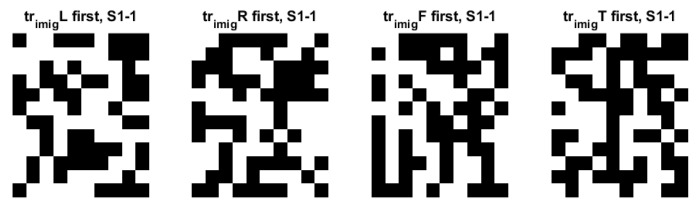
Training QR code realization of subject 1 at trial 1 for 4 classes: left, right, feet, and tongue (12 × 10) in C3D3a_4C.

Figure 10 shows the domain-wise similarity between the training and testing domains for subject 1. Considering their importance and the degree of information, only the QR images for subject 1 and trial 1 are shown among training or testing data and several trials. There are many similarities between training and testing QR images. We observed the trial-wise similarity of QR images between the first and last trials for subject 1. We also examined subject-wise similarity of QR images between subjects in the same first trial. It was confirmed that QR-EFA maximizes the three proposed similarities in domain, trials, and subjects.

**Figure 11:**
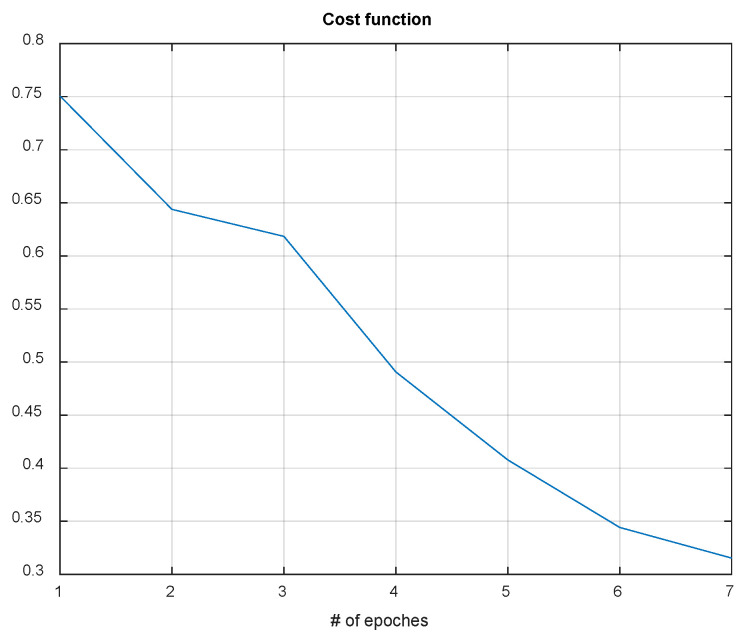
Data graph of the CNN cost function evolution vs. number of epochs in C3D3a_4C.

Using QR-EFA, a sample output of the CNN testing results for BCI competition III dataset 3a (C3D3a_4C) is shown in Figure 11. As the number of epochs increases during simulations, the cost or loss function decreases and reaches a limit.

The MATLAB code for a sample mean of C3D3a_4C data and its confidence interval, when the sample size n = 10 is, as follows (Box 3).

Box 3 **The MATLAB code for a sample mean of C3D3a_4C.**

% result set #1

x1(1) = 0.907143; x1(2) = 0.795238; x1(3) = 0.802381; x1(4) = 0.980952;

x1(5) = 0.919048; x1(6) = 0.859524; x1(7) = 0.921429; x1(8) = 0.904762;

x1(9) = 0.909524; x1(10) = 0.821429;

% result set #2

x1(1) = 0.857143; x1(2) = 0.930952; x1(3) = 0.928571; x1(4) = 0.926190;

x1(5) = 0.945238; x1(6) = 0.864286; x1(7) = 0.871429; x1(8) = 0.783333;

x1(9) = 0.928571; x1(10) = 0.952381;

SD1 = std(x1); % Standard deviation (SD)

SE1 = SD1/sqrt(length(x1)); % Standard error(SE)

ts1 = tinv([0.025 0.975],length(x1)−1);

% T-value/score for 95% CI (2.26)

CI1 = mean(x1) + ts1 * SE1;

% Confidence Intervals

>>mean(x1)

Ans = 0.8821

>>std(x1)

ans = 0.0602

>>CI1 % Confidence_Interval

CI1 = 0.8390 0.9252

>>CI1(2)-mean(x1) %Confidence Interval

Ans = 0.0431

The computation, simulation, verification, and validation on the proposed scheme requires considerations of the confidence interval for the given number of trials (*n*) and variance or standard deviations. Based on the nature of random seeds in statistical signal processing, we consider a 95% confidence interval, due its variance and spread for data augmentation, CNN layer initiations for kernel filters, and QR-eigenface selections for training images.

From the above results, with a 95% confidence interval, the sample mean was estimated to be 0.88, with a confidence interval of 0.0431. Note that, in this case, the Student’s *t*-distribution is applicable for calculating the confidence interval and sample variance (*s*^2^), because we do not have information regarding the variance or standard deviation (SD) of the population distribution in BCI datasets (C3D3a_4C or C4D2a_4C both). Table 3 shows the 10 simulation results, as the random seeds changes uniformly for statistically justification for confidence interval.

To calculate a 95% confidence interval for the sample mean *μ*, an estimated coefficient value 2.26 was used [35]. Thus, the mean and confidence interval of accuracy were 88.21% ± 6.02%, with the confidence interval of 4.31, i.e., (83.90, 92.52). Consequently, the probability for the same mean under the 95% confidence interval is given by Equation (8). For a comparison, the latest and best classification accuracy reported thus far for EEG four classes on BCI competition IV 2a was 85.4 [31].

(8) $$P\left(\left|\bar{X}-\mu \right|<2.26\sigma /\sqrt{n}\right)=P\left(\left|\frac{\bar{X}-\mu }{s/\sqrt{n}}\right|<2.26\right)=0.95$$

Then, we compute the confidence interval using Equation (9).

(9) $$\left[\bar{X}-2.26\frac{s}{\sqrt{n}},\bar{X}+2.26\frac{s}{\sqrt{n}}\right]=\left[83.90,92.52\right]$$

To validate and verify QR-EFA in a real dataset, with a comparison to C3D3a_4C, the next section is for the result of C4D2a_4C. Table 3 shows the number of trials per subjects for C4D2a_4C. The C4D2a_4C dataset is composed of nine subjects and the predefined number of experimental trials. The number of trials for left, right, foot, and tongue classes in the C4D2a_4C dataset, composed of nine subjects and the predefined number of experimental trials, was 72.

The detailed information of the property for C4D2a_4C is given as follows (Box 4).

Box 4 **The detailed information of the property for C3D2a_4C on MATLAB.**

comment1: ‘dataset: C4D2a_4C’

date: ‘2021.02.12’

madeby: ‘4C’

affiliation: ‘KNIT’

window: ‘offset: 3.5, length: 2’

subject: ‘subject #: 1’

prefiltering: ‘off’

s: 250 (sample/sec)

y: [1 × 288 double]

x: [500 × 22 × 288 double]

c: [22 × 1 cell]

Using the designated CNN model, which is depicted in Figure 7, MI classification EEG images extracted with QR-EFA were classified for C4D2a_4C. The accuracy was obtained for the test dataset of 2592 sheets, as shown in Table 4.

**Figure 12:**
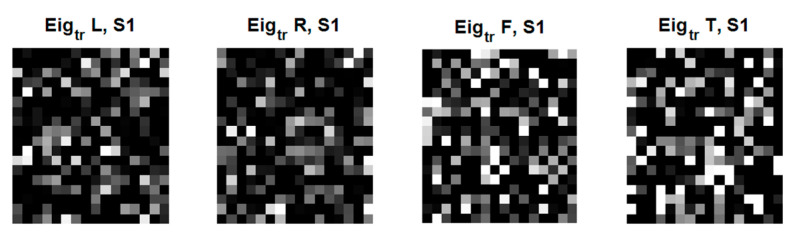
An original eigenface images of subjects for training data for 4 classes: left, right, feet, and tongue after whitening (18 × 16: = 72 × 4) in C4D2a_4C.

In Figure 12, the eigenfaces has been formulated. The eigenfaces reflecting the number of classes were selected. Considering a supervisory learning limitation, we designed the first to left, second, right, third to feet, and last to tongue classes.

**Figure 13:**
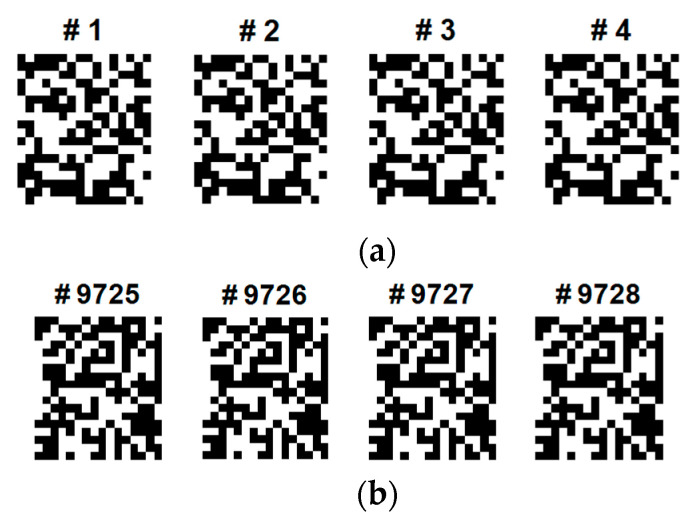
Some selected sample QR training images of data augmentation for the classes of (**a**) left and (**b**) tongue (18 × 16 in C4D2a_4C).

The training coefficients by projecting the training images to the QR-eigenfaces and testing coefficients by projecting the testing images to the QR-eigenfaces were obtained. The next step is to conceive of new QR images for the training and testing dataset. Afterwards, we will perform a liner combination of the QR-eigenface, depending on the relevant coefficients. The results of the linear combination were the QR images for the training and testing data. Figure 13 shows the results after brightness data augmentation in the brightness direction. Among four classes, the selected and data augmented images for left and tongue classes are shown.

**Figure 14:**
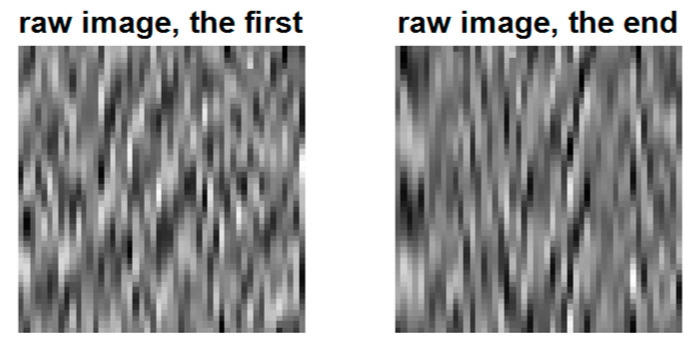
Primitive raw images for EEG data signal for the selected first and last trials (50 × 50) in C4D2a_4C.

Figure 14 shows the typical neuro raw images. The left image was of the first trial and the right was of the last trial for subject. First, we need to check the subject-wide similarity among subjects. Secondly, we check the domain-wise similarity between training and testing domain. Finally, we check the trial-wise similarity among trials.

**Figure 15:**
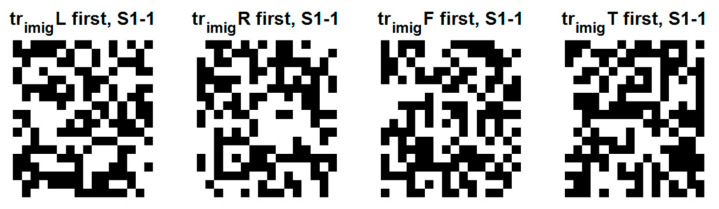
Training QR code realization of subject 1 at trial 1 for 4 classes: left, right, feet, and tongue (18 × 16) in C4D2a_4C.

Figure 15 shows the domain-wise similarity between training and testing domain for subject 1 and trial 1. There were also similarities between the training and testing images. Hence, we also maximized the similarities in the directions of domain, trials, and subjects.

**Figure 16:**
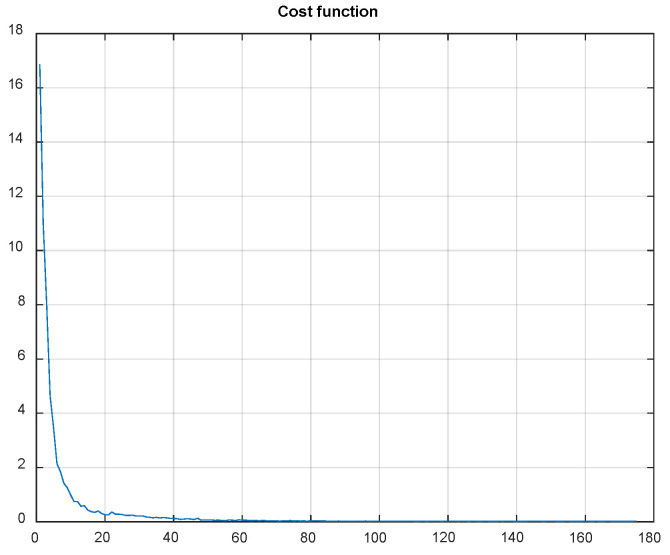
Data graph of the CNN cost function evolution vs. number of epochs in C4D2a_4C.

In summary, it is clear that QR-EFA guarantees a distinguishable and discriminative feature per class, as well as a higher similarity, with respect to domain-, trial-, and subject-wise similarities. This unique novel scheme accelerates the improved accuracy in CNNs. With QR-EFA, a sample output of CNN testing results for in C4D2a_4C is provided in Figure 16. It also shows that the cost function reached the limit.

The MATLAB code for a sample mean of in C4D2a_4C data and its confidence interval, when the sample size n = 10 is, as follows (Box 5).

Box 5 **The MATLAB code for a sample mean of in C4D2a_4C data.**

% result set #1

x1(1) = 0.966435; x1(2) = 0.976852; x1(3) = 0.984954; x1(4) = 0.971065;

x1(5) = 0.969136; x1(6) = 0.983025; x1(7) = 0.986883; x1(8) = 0.985725;

x1(9) = 0.984182; x1(10) = 0.978395;

SD1 = std(x1); % Standard deviation (SD)

SE1 = SD1/sqrt(length(x1)); % Standard error(SE)

ts1 = tinv([0.025 0.975],length(x1)−1); % T-value/score for 95% CI (2.26)

CI1 = mean(x1) + ts1 * SE1; % Confidence Intervals

>>mean(x1)

Ans = 0.9787

>>std(x1)

ans = 0.0075

>>CI1 % Confidence_Interval

CI1 = 0.9733 0.9840

>>CI1(2)-mean(x1) %Confidence Interval

Ans = 0.0054

Table 5 shows the 10 simulation results, as the random seeds changes uniformly for statistically justification for confidence interval.

From the above results, with a 95% confidence interval, we obtained a range of estimates for a sample mean (0.98), and the confidence interval was 0.0054. The mean and confidence interval of the accuracy was 97.87 ± 0.0075%, with a confidence interval of 0.0054, i.e., (97.33, 98.40). The latest and best classification result is shown below. Then, we compute the confidence interval using Equation (10).

(10) $$\left[\bar{X}-2.26\frac{s}{\sqrt{n}},\bar{X}+2.26\frac{s}{\sqrt{n}}\right]=\left[97.33,98.40\right]$$

## 5. Conclusions

In this study, we proposed the QR-EFA method for efficient motor-imaginary (MI) EEG classification and showed that the EEG signal, when converted into a standardized and sharable QR image, can be classified when using a simple CNN. To obtain a unique feature per class, EEG signal data can be utilized with the concepts of domain-, trial-, and subject- similarities. The EEG data measured from several subjects were used using the BCI competition 3 dataset 3a (C3D3a_4C) and BCI competition 4 dataset 2a (C4D2a_4C). Through QR-EFA method, the EEG signal was converted into EEG QR image, as formed with the EFA method, and a data augmentation technique was applied to solve the limited EEG image problem.

For optimum and best classification performance, QR-EFA maximizes the proposed domain-, trial-, and subject-wise similarities. As far as we are concerned, none of the literature in BCIs has considered the domain-, trial-, and subject-wise similarities so far. Based on this observation, our proposed QR-EFA method was used to maximize the three similarities in the directions of domains, trials, and subjects. Using a simple seven-layer CNN model, data classification results showed an exceptional and remarkable accuracy of 97.87% ± 0.75, with a confidence interval of 0.54, i.e., (97.33, 98.40) for the C4D2a_4C and 88.21% ± 6.02, with a confidence interval of 4.31, i.e., (87.90, 92.52) for the C3D3a_4C. Unlike the CSP method, which is only robust in the original two-class classification, the proposed QR-EFA method is applicable to the classification of two multi-classes of EEG signal; hence, it definitely advantages from the use of the QR-EFA algorithm. Because the QR-EFA method extracts classification features and generates EEG QR images that are well-differentiated between classes, it is suitable as training and testing input images for further ML process and can greatly contribute to classification performance improvement.

Because the ML technique for image classification is becoming stronger with the development of better performance models and hardware performance, QR-EFA’s flexible EEG conversion method or frameworks, which should be applied to non-motor imaginary (MI), such as word thinking, emotion detections, arithmetic calculation operations, and multi-class classification, will be developed in the future. The application to a larger number of classes, such as five or six categories, will be applied, with slight extensions in time.

## Figures and Tables

**Table 1:** The C3D3a_4C dataset composed of three subjects and predefined number of experimental trials.

Subject	Class (# of Trials)			
	Left (L)	Right (R)	Foot (F)	Tongue (T)
1	45	45	45	45
2	30	30	30	30
3	30	30	30	30

**Table 2:** EEG analysis accuracy for C3D3a_4C using QR-EFA method.

		Subjects			
		A1	A2	A3	Average
Accuracy (%)	EFA_LDA, two classes	52.22	46.67	63.33	54.07
	QR-EFA	90.00	93.33	90.83	91.11

**Table 3:** Accuracy results of classification for 10 simulations, with changing random seeds uniformly in C3D3a_2C.

# of Simulations	Subject (Failed/Trials)			Success Rate
	1	2	3	
1	26/180	15/120	19/120	0.857143
2	16/180	10/120	3/120	0.930952
3	11/180	13/120	6/120	0.928571
4	9/180	17/120	5/120	0.926190
5	5/180	12/120	6/120	0.945238
6	30/180	14/120	13/120	0.864286
7	27/180	9/120	18/120	0.871429
8	42/180	32/120	17/120	0.783333
9	10/180	15/120	5/120	0.928571
10	18/180	0/120	2/120	0.952381

**Table 4:** EEG analysis accuracy for C4D2a_4C, using QR-EFA method.

		Subjects									
		1	2	3	4	5	6	7	8	9	Average
Accuracy (%)	EFA_LDA, two classes	53.47	52.08	55.55	55.55	54.16	45.13	58.33	47.72	51.38	52.55
	QR-EFA	97.15	97.99	97.05	98.33	98.47	97.85	98.78	97.36	97.81	97.87

**Table 5:** Accuracy results of classification for 10 simulations, with changing random seeds uniformly in C4D2a_2C.

# of Simulations	Subject (Failed/Trials)									Success Rate
	1	2	3	4	5	6	7	8	9	
1	18/288	6/288	19/288	9/288	7/288	6/288	2/288	13/288	7/288	0.966435
2	7/288	9/288	3/288	5/288	4/288	8/288	6/288	6/288	12/288	0.976852
3	2/288	2/288	10/288	1/288	6/288	5/288	0/288	4/288	9/288	0.984954
4	19/288	8/288	10/288	7/288	1/288	9/288	7/288	8/288	6/288	0.971065
5	11/288	5/288	10/288	13/288	10/288	12/288	5/288	10/288	4/288	0.969136
6	6/288	4/288	7/288	5/288	3/288	2/288	2/288	8/288	7/288	0.983025
7	3/288	5/288	3/288	1/288	3/288	7/288	4/288	6/288	2/288	0.986883
8	3/288	4/288	5/288	0/288	3/288	4/288	7/288	5/288	6/288	0.985725
9	1/288	8/288	9/288	3/288	3/288	4/288	0/288	9/288	4/288	0.984182
10	12/288	7/288	9/288	4/288	4/288	5/288	2/288	7/288	6/288	0.978395

## Data Availability

The data presented in this study are included within the article.

## Abbreviations

The following abbreviations are used in this manuscript:

EFA	Eigenface analysis
QR-EFA	Quick eigenface analysis
BCI	Brain computer interface
EEG	Electroencephalogram
CSP	Common spatial pattern
C3D3a_4C	BCI competition III dataset IIIa for four classes
C4D2a_4C	BCI competition IV dataset IIa for four classes
PCA	Principal component analysis
ICA	Independent component analysis

## References

[1] Classification Ranking for EEG 4 Classes on BCI Competition IV 2a. https://paperswithcode.com/sota/eeg-4-classes-on-bci-competition-iv-2a.

[2] Pfurtscheller G., Neuper C., Guger C., Harkam W., Ramoser H., Schlogl A., Obermaier B., Pregenzer M. (2000). Current trends in Graz brain-computer interface (BCI) research. IEEE Trans. Neural Syst. Rehabil. Eng..

[3] Schalk G., McFarland D.J., Hinterberger T., Birbaumer N., Wolpaw J.R. (2004). BCI2000: A general-purpose brain-computer interface (BCI) system. IEEE Trans. Biomed. Eng..

[4] Belwafi K., Gannouni S., Aboalsamh H. (2020). An Effective Zeros-Time Windowing Strategy to Detect Sensorimotor Rhythms Related to Motor Imagery EEG Signals. IEEE Access.

[5] Nam C.S., Nijholt A., Lotte F. (2018). Brain–Computer Interfaces Handbook: Technological and Theoretical Advances.

[6] Boi F., Moraitis T., De Feo V., Diotalevi F., Bartolozzi C., Indiveri G., Vato A. (2016). A bidirectional brain-machine interface featuring a neuromorphic hardware decoder. Front. Neurosci..

[7] Chin-Teng L., Yu-Chieh C., Teng-Yi H., Tien-Ting C., Li-Wei K., Sheng-Fu L., Hung-Yi H., Shang-Hwa H., Jeng-Ren D. (2008). Development of Wireless Brain Computer Interface with Embedded Multitask Scheduling and its Application on Real-Time Driver’s Drowsiness Detection and Warning. IEEE Trans. Biomed. Eng..

[8] Nicolas-Alonso L.F., Gomez-Gil J. (2012). Brain Computer Interfaces, a Review. Sensors.

[9] Jinyi L., Yuanqing L., Hongtao W., Tianyou Y., Jiahui P., Feng L. (2012). A Hybrid Brain Computer Interface to Control the Direction and Speed of a Simulated or Real Wheelchair. IEEE Trans. Neural Syst. Rehabil. Eng..

[10] Lemm S., Blankertz B., Curio G., Muller K. (2005). Spatio-spectral filters for improving the classification of single trial EEG. IEEE Trans. Biomed. Eng..

[11] Aznan N.K.N., Yeon-Mo Y. Applying Kalman filter in EEG-Based Brain Computer Interface for Motor Imagery classification. Proceedings of the 2013 International Conference on ICT Convergence (ICTC).

[12] Lotte F., Congedo M., Lécuyer A., Lamarche F., Arnaldi B. (2007). A review of classification algorithms for EEG-based brain–computer interfaces. J. Neural Eng..

[13] Aznan N.K.N., Huh K.-M., Yang Y.-M. (2016). EEG-based motor imagery classification in BCI system by using unscented Kalman filter. Int. J. Inf. Commun. Technol..

[14] Roijendijk L., Gielen S., Farquhar J. (2016). Classifying Regularized Sensor Covariance Matrices: An Alternative to CSP. IEEE Trans. Neural Syst. Rehabil. Eng..

[15] Robinson N., Vinod A.P., Kai Keng A., Keng Peng T., Guan C.T. (2013). EEG-Based Classification of Fast and Slow Hand Movements Using Wavelet-CSP Algorithm. IEEE Trans. Biomed. Eng..

[16] Husain A.M., Sinha S.R. (2017). Continuous EEG Monitoring: Principles and Practice.

[17] Jin J., Xiao R., Daly I., Miao Y., Wang X., Cichocki A. (2020). Internal feature selection method of CSP based on L1-norm and Dempster–Shafer theory. IEEE Trans. Neural Netw. Learn. Syst..

[18] Blankertz B., Muller K., Curio G., Vaughan T.M., Schalk G., Wolpaw J.R., Schlogl A., Neuper C., Pfurtscheller G., Hinterberger T. (2004). The BCI competition 2003: Progress and perspectives in detection and discrimination of EEG single trials. IEEE Trans. Biomed. Eng..

[19] Vidaurre C., Krämer N., Blankertz B., Schlögl A. (2009). Time Domain Parameters as a feature for EEG-based Brain–Computer Interfaces. Neural Netw..

[20] Lotte F., Guan C. (2010). Regularizing common spatial patterns to improve BCI designs: Unified theory and new algorithms. IEEE Trans. Biomed. Eng..

[21] Choi H., Park J., Lim W., Yang Y.-M. (2021). Active-beacon-based driver sound separation system for autonomous vehicle applications. Appl. Acoust..

[22] Yu X., Chum P., Sim K.-B. (2014). Analysis the effect of PCA for feature reduction in non-stationary EEG based motor imagery of BCI system. Optik.

[23] Pearson K. (1901). LIII. On lines and planes of closest fit to systems of points in space. Lond. Edinb. Dublin Philos. Mag. J. Sci..

[24] Lu H., Eng H.-L., Guan C., Plataniotis K.N., Venetsanopoulos A.N. (2010). Regularized common spatial pattern with aggregation for EEG classification in small-sample setting. IEEE Trans. Biomed. Eng..

[25] Bengio Y., Goodfellow I., Courville A. (2017). Deep Learning.

[26] Wang Y., Huang G., Song S., Pan X., Xia Y., Wu C. (2021). Regularizing deep networks with semantic data augmentation. IEEE Trans. Pattern Anal. Mach. Intell..

[27] Lee J., Won K., Kwon M., Jun S.C., Ahn M. (2020). CNN with large data achieves true zero-training in online P300 brain-computer interface. IEEE Access.

[28] The BCI Competition IV Dataset 2a for Four Classes (C4D2a_4C) BCI-Competition III (2008). https://www.bbci.de/competition/iv.

[29] The BCI Competition III Dataset 3a for Four Classes (C3D3a_4C) BCI-Competition III (2005). https://www.bbci.de/competition/iii.

[30] He L., Hu D., Wan M., Wen Y., von Deneen K.M., Zhou M. (2016). Common Bayesian network for classification of EEG-based multiclass motor imagery BCI. IEEE Trans. Syst. Man Cybern. Part A Syst. Hum..

[31] Kim K.M., Choe S.-H., Ryu J.-M., Choi H. (2020). Computation of Analytical Zoom Locus Using Padé Approximation. Mathematics.

[32] Skansi S. (2018). Introduction to Deep Learning: From Logical Calculus to Artificial Intelligence.

[33] Kim J., Ko J., Choi H., Kim H. (2021). Printed Circuit Board Defect Detection Using Deep Learning via A Skip-Connected Convolutional Autoencoder. Sensors.

[34] Riaz H., Park J., Choi H., Kim H., Kim J. (2020). Deep and Densely Connected Networks for Classification of Diabetic Retinopathy. Diagnostics.

[35] Jung U., Choi H. (2022). Active echo signals and image optimization techniques via software filter correction of ultrasound system. Appl. Acoust..

